# Late surgical ventricular septal defect closure in a low middle-income country setting: a case series

**DOI:** 10.1186/s13256-023-03972-4

**Published:** 2023-06-01

**Authors:** Zawadi Edward Kalezi, Naizihijwa Majani, Alphonce Nsabi Simbila, Stella Mongella, Godwin Godfrey Sharau, Deogratias Nkya, Sulende Kubhoja

**Affiliations:** 1 Jakaya Kikwete Cardiac Institute, P.O Box 65141, Dar es Salaam, Tanzania; 2 Emergency Medicine Department, Muhimbili National Hospital, Dar es Salaam, Tanzania; 3 Muhimbili University of Health and Allied Sciences, Dar es Salaam, Tanzania

**Keywords:** Ventricular septal defect, Pulmonary hypertension, Congenital heart disease, Tanzania

## Abstract

**Background:**

Ventricular septal defect (VSD) is the commonest type of congenital heart lesion accounting for up to 40% of congenital heart defects. Well timed VSD closures are reported to yield excellent long-term outcomes. Late surgical VSD closures, particularly from the developing countries, are infrequently reported.

**Case presentation:**

We report three cases of African children aged between 13 and 14 years who had late VSD presentations. They reported complaints of growth failure and recurrent respiratory infections since early infancy which necessitated frequent visits to primary health care facilities. They were found to have large ventricular septal defects by thoracic echocardiography. Diagnostic cardiac catheterization was done to all three patients to rule out irreversible pulmonary hypertension. After promising cardiac catheterization findings, they all underwent successful surgical VSD repair with good early outcomes.

**Conclusion:**

VSD surgical closure is ideal in children below 2 years, however, it can be done in children who present at advanced age despite being considered high risk patients. All three of our patients who presented late had successful surgical VSD repairs with promising immediate outcome. The role of genetics in the protection against developing irreversible pulmonary vascular disease in these patients is a possible area for future studies.

## Background

The incidence of congenital heart disease (CHD) varies in different studies, ranging from 6 to 75 per 1000 live births [1]. Ventricular septal defect (VSD) is regarded as a common congenital heart lesion, second in prevalence to bicuspid aortic valve [2, 3]. The global incidence of VSD is reported to be 1.5 to 3.5 per 1000 live births [1].

The location and size of the VSD markedly determine the spontaneous closure rate and clinical spectrum defects. Small defects often close spontaneously by two years of age, however, larger defects and those involving inlet and outlet septum usually require surgical intervention [3, 4]. Peri-membranous defects take longer than muscular defects to close spontaneously. Nevertheless, peri-membranous and doubly committed VSDs have been associated with aortic regurgitation, thereby necessitating early surgical intervention [3, 5].

The natural history of haemodynamic significant VSDs has been studied in a cohort of patients. Morbid events that occurred during the follow-up period included bacterial endocarditis, congestive heart failure, brain abscess, stroke and pulmonary hypertension [3]. Pulmonary hypertension should be closely monitored due to the risk of irreversible pulmonary vascular disease. Long-term complications of unrepaired VSD are Eisenmenger syndrome (ES), right ventricular failure, and early death [3, 6]. Nearly 50% of all patients with unrepaired large VSDs are at risk of developing ES at one point in time [7].

Surgery is the treatment of choice during early childhood to avoid consequences of haemodynamic significant VSD. Surgical closure of VSD is indicated for children less than 2 years of age with poorly controlled symptoms of heart failure, growth failure, elevated but responsive pulmonary vascular resistance, or recurrent chest infections requiring hospitalization [3].

Timing of cardiac surgery is of paramount importance due to complications accompanying late VSD closure. Surgery is of high risk when ventricular dysfunction or pulmonary vascular disease sets in [3, 6]. The time for cardiac surgical closure is reported to be late in developing countries compared to the developed world [6]. In Nigeria for example, 30.7% of children with detected cardiac anomalies had surgical closure after their fifth birthday, with a maximum age of 18 years [6]. Conversely, in California, the mean age at VSD closure was reported to be 2.8 years over a period of one year [6].

Regardless of proper timing of surgery, complications following surgical VSD closure tend to be more common than mortality [3]. Reported early complications include residual VSD, post pericardiotomy syndrome, patch dehiscence and arrhythmias [3, 5, 8]. Cardiac arrhythmias such as right bundle branch block (RBBB) and sinus node dysfunction are quite common after surgical VSD closure [3, 5]. In Sweden, 8 out of 22 children who had VSD closure developed right bundle branch block [9]. Symptomatic bradycardia was reported in 13% of patients who were followed over a decade after VSD surgical closure [8].

We report on three late presenting patients with VSD who underwent successive surgical VSD closure.

## Case presentations

### Patient 1

A 13- year-old African female from upcountry presented with awareness of heartbeat and easy fatigue on mild exertion. The patient’s father reported that she had been unwell since early infancy with recurrent chest infections and poor weight gain. She was diagnosed with a VSD at the age of 12 years but delayed seeking further care due to financial constraints. She was initiated on diuretics and angiotensin-converting enzyme (ACE) inhibitors. On arrival the patient weighed 22.6 kg, pulse rate was 96 beats/min, respiratory rate was 23 breaths/min, a blood pressure of 108/57 mmHg, and oxygen saturation of 97% on room air. On cardiovascular examination, the patient was noted to have a laterally displaced apex beat and a grade 4/6 holosystolic murmur at the left lower sternal border. Echocardiogram (ECHO) revealed hyperdynamic left ventricular function with a large Peri-membranous VSD measuring 16 mm shunting left-to-right. Diagnostic cardiac catheterization revealed severe pulmonary hypertension (mean pulmonary artery pressure more than 2/3 systemic artery pressure) with normal pulmonary vascular resistance. The mean pulmonary artery pressure was 51 mmHg on room air and 40 mmHg on 100% oxygen. The pulmonary vascular resistance was 1 and 0.48 Wood unit/m^2^ on room air and on 100% oxygen respectively. She thereafter underwent VSD closure through right atriotomy with an inter-atrial communication created at fossa ovale to act as a pop-up valve in case of pulmonary hypertensive crisis. She had uneventful intraoperative course and was extubated on the same day. Immediate post-surgical echocardiography showed an intact VSD patch with residual shunt through and a pressure gradient of 60 mmHg. Immediate post-surgery treatment consisted of inotropes and anti-failure medications. On the second day post-surgery, an oral pulmonary vasodilator was added for pulmonary hypertension. She developed junctional ectopic tachycardia on the third day post-surgery which responded to oral Propranolol. She spent five days in the intensive care unit (ICU) and was later transferred to the normal ward with an improved left ventricular function, ejection fraction of 60%. Two weeks post-surgery, she was discharged home. Due to financial constraints, she couldn’t attend a follow-up clinic. However, 9 months post-surgery her father reported she was doing well through a telephone consultation.

### Patient 2

A 14- year-old African male with history of difficulty in breathing and failure to thrive since infancy presented with exertional fatigue. He was diagnosed with a VSD at the age of 11 months and received treatment with diuretics and angiotensin-converting enzyme inhibitors. He attended regular follow-up cardiac clinics but stopped at the age of 3 years due to financial constraints. On admission, he weighed 30 kg with a pulse rate of 88 beats/min, respiratory rate of 18 breaths/min, and oxygen saturation of 95% on room air. On cardiovascular examination, he had a laterally displaced apex beat and a grade 3/6 holosystolic murmur at the left lower sternal border, and a pronounced second heart sound. His transthoracic echocardiography revealed a dilated left heart, severe mitral regurgitation, and a large subpulmonic VSD measuring 12 to 14 mm withe bidirectional flow; LVIDd/s 6.0 cm/4.0 cm, IVsd 1.5 cm, LVPWd 1.2 cm and ejection fraction of 62%. Diagnostic cardiac catheterization revealed a pulmonary vascular resistance of 3.15 and 1.4 Wood unit/m^2^ on room air and on 100% oxygen respectively. Mean pulmonary arterial pressure was 51 mmHg on room air and 54 mmHg on 100% oxygen. Through the right atriotomy, he underwent a VSD closure with a pop-up valve created in case of pulmonary hypertensive crisis three weeks after a diagnostic cardiac catheterization. His intraoperative course was uneventful and was extubated on the same day. Immediate transthoracic echocardiogram post-surgery showed an intact VSD patch without residual shunt. There were mild mitral regurgitation and tricuspid regurgitation. LVIDd/s 5.9 cm/4.0 cm, IVsd 1.4 cm, LVPWd 1.0 cm and ejection fraction of 60%. An oral pulmonary vasodilator was added on the second day post- surgery for pulmonary hypertension. While in ICU, on the fourth day post-surgery, he developed premature ventricular contractions (PVC) which were controlled by oral Amiodarone. He was later discharged 2 weeks post-surgery with diuretics, ACE inhibitor and on tapering dose of oral Amiodarone. A nine-month follow-up ECHO showed mild tricuspid regurgitation (TR), but no residual VSD was seen (Table 1**)**. His electrocardiogram (ECG) showed a first-degree heart block; however, no symptoms were reported. For the asymptomatic heart block and increased pulmonary vascular resistance, regular follow-up was deemed necessary.

**Table 1:** Follow- up Echocardiographic parameters

	Patient 2	Patient 3
Residual VSD	None	None
LV end systolic dimension (mm)	14	15
LV end diastolic dimension (mm)	59	47
LV fractional shortening (%)	33	32
Aortic regurgitation	None	None
Mitral regurgitation	Mild	None
Pulmonary regurgitation	None	None
Tricuspid regurgitation, pressure gradient (mmHg)	Mild [8]	Trivial [10]

LV; Left ventricle, VSD; Ventricular septal defect

### Patient 3

A 13- year-old African male presented with recurrent chest infections since early childhood. Diagnosis of VSD was made at the age of 8 months but defaulted regular follow-up due to a long travel distance to health facility and financial constraints. On arrival he weighed 22.6 kg, had a pulse rate of 100 beats/min, respiratory rate was 24 breaths/min, a blood pressure of 98/60 mmHg, and oxygen saturation of 98% on room air. On examination, he had a laterally displaced apex beat and a grade 3/6 holosystolic murmur at the left lower sternal border, and a loud second heart sound. Echocardiogram showed dilated left atrium and ventricle with a large Peri-membranous VSD. He underwent diagnostic cardiac catheterization that revealed a pulmonary vascular resistance of 4.8 and 2.4 wood unit on room air and on 100% oxygen respectively. Mean pulmonary arterial pressure was 50 mmHg on room air and 56 mmHg on 100% oxygen. He underwent a successful VSD repair with an uneventful intraoperative and immediate postoperative course. He also had a pop- up valve in case of pulmonary hypertension crisis like the other cases described previously. On the same day post-surgery, the patient was extubated uneventfully and was started on oral pulmonary vasodilator. He stayed in the ICU for a total of 4 days with no encountered complications. He was discharged 8 days later to attend a routine cardiology follow up clinic. A seven-month follow-up ECHO showed no residual shunt across the VSD patch but had a trivial tricuspid regurgitation (Table 1**)**. LVIDd/s 4.7 cm/3.3 cm, IVsd 1.5 cm, LVPWd 1.1 cm and an ejection fraction of 58%.

## Discussion

Surgical closure is the primary treatment strategy in children with large ventricular septal defects (VSD). It is indicated in children less than 2 years of age with poorly controlled symptoms of congestive heart failure, growth failure, elevated but responsive pulmonary vascular resistance, or recurrent chest infections. Various studies have reported generally excellent outcomes following early-surgical VSD closure, however, there is scarcity of data regarding late surgical closure in children [3]. Nevertheless, few studies have been documented in developing countries [6].

We presented a series of three patients aged between 13 and 14 years with late VSD presentation. These patients presented with long-standing histories of growth failure and recurrent respiratory infections necessitating frequent hospitalizations at peripheral health facilities. These clinical features were typical indicators for surgery, however, such reasons as financial constraints and long distances needed to be travelled to reach appropriate facilities hindered accessibility of timely surgical treatment. These eventually led to late hospital presentation and hence late surgical VSD repair. Nonetheless, our facility is the only cardiac centre which offers surgical services in children, and it is situated far from most of these patients. Noteworthy, lack of awareness of both parents and primary health care providers on congenital heart diseases mighty have contributed to the late presentation.

There were anticipated long-term haemodynamic consequences brought about by delayed surgical treatment in these patients, therefore, diagnostic cardiac catheterization was needed to determine the optimal management strategy while accounting for risk factors [3]. Surprisingly, diagnostic catheterization showed promising results which aided the decision to undergo complete repair at this advanced age. The explanation for vasoreactive pulmonary vascular resistance at this advanced age was challenging to extrapolate. Nonetheless, one of our patients, was found to have normal pulmonary vascular resistance.Genetic predisposition has been mentioned as one among the reasons to why some patients are at the greatest risk of developing pulmonary vascular resistance [7].

Two of our patients developed arrhythmias immediately post-surgery which were medically controlled. Arrhythmias have been reported to occur even in children who have been operated early, however, majority of them are transient and resolve without treatment [3], similarly reported by Menting *et al.* [8]*.* The causes of arrythmias could be the cannulation of the right atrium for cardiopulmonary bypass [5] or direct injury to the right branch of the bundle of His during surgery [3]. Additionally, right heart strain which signifies right ventricular dysfunction is often associated with supraventricular tachycardia [10]. Right ventricular dysfunction resulting from pulmonary arterial hypertension, can be characterized by dilated right atrium, flattening of interventricular septum, marked tricuspid regurgitation, or dilated right ventricle which were not found on our patients.

Long-term surveillance to assess for arrythmias is indicated for children who have undergone surgical VSD closure regardless of the age at surgery [3]. Two of our patients developed arrhythmias immediately post-surgery, even though one patient is lost to follow-up, but the other one is asymptomatic. Patients who have delayed surgical VSD closure are at increased risk for arrythmias even long after the surgical procedure including symptomatic PVCs and impaired heart rate variability presenting during adulthood [11]. Despite good early outcomes in the setting of these patients who underwent late surgical closure, long-term follow-up is highly recommended to assess arrhythmia and management of any other untoward events.

Routinely at our institute, children who have undergone surgical VSD closure are discharged within a week postoperatively. In this set of patients, the average hospital stay was 14 days which is contrary to what has been expected in view of the accompanying complications with late surgical closure.

## Conclusion

Surgical VSD closure is ideal in children below 2 years. It can however be done in children who present at advanced age with promising immediate outcomes despite being considered high risk patients. Favourable diagnostic cardiac catheterization findings need to be appreciated beforehand to inform the decision to perform surgical intervention. Further studies are needed to look at the possibility of genetic protection against developing irreversible pulmonary vascular disease.

## Data Availability

Not applicable.

## Abbreviations

ACE: Angiotensin converting enzyme
CHD: Congenital heart disease
ECHO: Echocardiography
ECG: Electrocardiography
ICU: Intensive care unit
LVID: Left ventricular internal dimension
PH: Pulmonary hypertension
PVC: Premature ventricular contraction
PVD: Pulmonary vascular disease
VSD: Ventricular septal defect
